# Surface Tension-Based Alignment of Microfibers on Hydrophilic–Superhydrophobic Grooved Surfaces

**DOI:** 10.3390/mi11110973

**Published:** 2020-10-29

**Authors:** Bo Chang, Jialong Jin, Quan Zhou

**Affiliations:** 1 College of Mechanical and Electrical Engineering, Shaanxi University of Science and Technology, Xi’an 710021, China; 1905004@sust.edu.cn; 2 School of Electrical Engineering, Aalto University, FI-00076 Aalto, Finland; quan.zhou@aalto.fi

**Keywords:** micro assembly, self-alignment, surface tension, hydrophilic–superhydrophobic, microfibers, orderly distribution, robotic micro assembly, micro manipulation, micro groove

## Abstract

Alignment and orderly distribution of microfibers have a major effect on the mechanical, electrical, and thermal properties of the fiber reinforced materials, biomimetic materials, and soft microsensors. However, it is still a challenging task to precisely align and distribute microfibers and construct complex patterns. This paper proposes a surface tension-based method to align and orderly distribute microfibers. A model was developed to simulate the surface tension driven alignment of the microfiber. We designed and fabricated hydrophilic–superhydrophobic grooved surfaces. We demonstrated that the microfibers can self-align to the hydrophilic–superhydrophobic grooves with different geometries. We studied the influence of the volume of the droplet and bias on the alignment success rate. The results indicate that the process can tolerate large variations of the bias and the volume, unless the volume is not enough to cover the groove. We further investigated the influence of the width of the groove on the alignment accuracy. The results show that the alignment accuracy is largely depending on the size difference between the groove and the microfiber; the better the size of the groove matches the size of the fiber, the higher the alignment accuracy will be achieved. The proposed method has great potential in construction of complex microstructures using microfibers.

## 1. Introduction

The distribution and the alignment of microfibers have a major effect on the mechanical [1], electrical [2], and thermal properties [3] of the fiber reinforced materials. Polymers with aligned carbon nanotubes have shown much higher conductivity and tensile strength compared to the polymer with non-aligned carbon nanotubes [4]. It has been reported that the tensile strength of an epoxy composite with aligned carbon nanotubes has improved from 8 MPa to 21.1 MPa and Young’s modulus from 415 MPa to 843 MPa [5]. It has been also shown that the electrical resistivity of aligned nanotubes was reduced by one order of magnitude compared to non-aligned nanotubes [6].

Many methods have been developed to align microfibers and form certain patterns, including electrospinning [7,8,9,10,11], 3D printing [12,13,14], robotic micromanipulation [15,16,17], microfluidics [18,19], and so on. Electrospinning uses electric force to dispense charged threads of polymer solutions or melt polymers and the method is especially suitable for producing exceptionally fine fibers such as micro and nanofibers. Distribution and alignment of micro- and nanofibers have been previously demonstrated using the electrospinning method [8,13], e.g., fibers with a certain geometry such as lattice pattern [13] and ivy shoot like patterns [20] have been achieved by adjusting the concentration of the polymer solution, flow rate, and electric field. A distribution of microfibers with the orientation of 90° and 60° has been achieved by combining 3D printing technique and melt electrospinning technique [7]. Microfluidics and robotic micromanipulation technology have also been reported to be able to align microfibers and construct simple structures, e.g., small amount of the microfibers can be distributed in parallel or orthogonally [18,19]. Despite the impressive results achieved so far, it is still challenging to precisely control the spacing of two individual microfibers and the orientation of each microfiber and construct more complex patterns. Previously, our research group has reported a hybrid microassembly technique [21,22,23,24,25] which combines the robotic micro-assembly technique and the surface tension-based self-alignment technique [26,27,28,29,30]. The hybrid microassembly technique has been demonstrated to be able to achieve accurate and fast alignments of microchips, achieving the assembly of 40,000 microchips per hour, with an accuracy higher than 1 µm, and a success rate of 98% [31]. The hybrid microassembly method has shown good potentials for construction of 3D microstructures; however, the method has not been investigated for the alignment of microfibers.

In this paper, we propose a surface tension-based method to align and orderly distribute microfibers. To understand the mechanism, a theoretical model was developed to simulate the alignment of the microfiber and analyze the driven force for the alignment of microfibers. We designed and fabricated grooved hydrophilic–superhydrophobic patterned surfaces. We demonstrated that the microfibers can self-align to the grooved hydrophilic–superhydrophobic patterned surfaces with different geometries. We studied the influence of the volume of the droplet and bias on the alignment success rate through systematic experiments. We further investigated the influence of the width of the grooved pattern on the alignment accuracy.

## 2. Materials and Methods

### 2.1. Alignment Strategy

**Figure 1:**
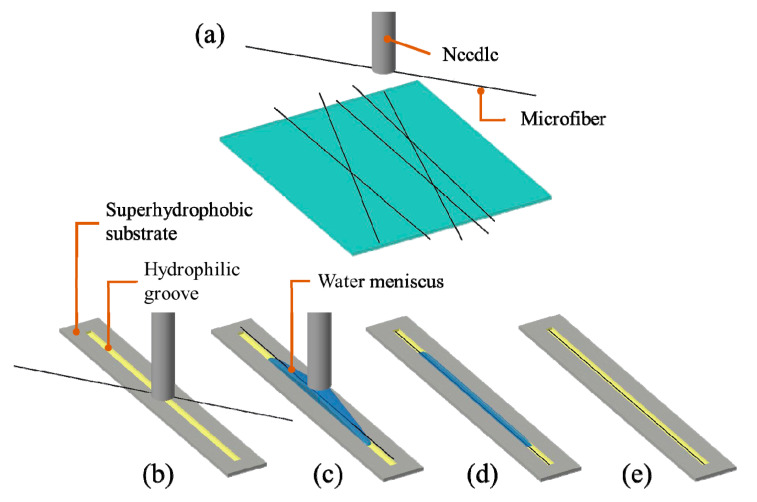
Schematic of surface tension-based alignment of a microfiber: (**a**) A microfiber is picked up by a needle; (**b**) The fiber is transport it to a target hydrophilic–superhydrophobic grooved surface; (**c**) The needle dispenses a drop of water and the water droplet is confined inside the hydrophilic groove; (**d**) The fiber is released from the needle and aligned to the groove; (**e**) The water droplet inside the groove evaporates.

In this paper, we propose a surface tension-based alignment method which combines surface tension assisted pick-and-place technique and hydrophilic–superhydrophobic grooved surfaces. It utilizes the surface tension assisted pick-and-place technique for the coarse positioning of the flexible fibers and applies hydrophilic–superhydrophobic grooved surfaces to achieve fine alignment of microfibers. The schematic of the alignment method is illustrated in Figure 1. Firstly, a microfiber is picked up by a dispensing needle with a droplet (Figure 1a); next, the fiber is transported to a target hydrophilic–superhydrophobic grooved surface (Figure 1b); and then the needle dispenses a drop of water and the water droplet is confined inside the hydrophilic groove and form a water meniscus (Figure 1c); next, the fiber is released from the needle and aligned to the target groove (Figure 1d); finally, the water droplet inside the groove evaporates leaving the fiber aligned to the groove (Figure 1e).

### 2.2. Fabrication of Hydrophilic–Superhydrophobic Grooved Surfaces

**Figure 2:**
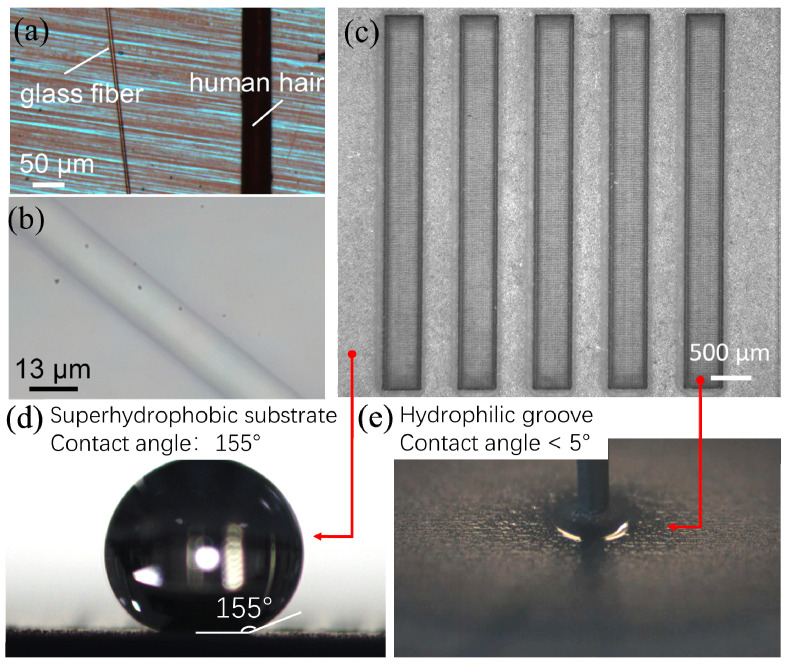
Glass fiber and grooved silicon substrate: (**a**) Microscopic view of a glass fiber next to a human hair; (**b**) Microscopic view of glass fiber with a diameter of 13 µm; (**c**) Hydrophilic–superhydrophobic patterned substrate with five parallel grooves; (**d**) Contact angle of water on the superhydrophobic substrate; (**e**) Contact angle of water in the hydrophilic groove.

To fabricate hydrophilic–superhydrophobic grooved surfaces for alignment of microfibers, superhydrophobic coating (WHOLE-NANO SPN-62, WHOLE-NANO Ltd, Suzhou, China) was firstly sprayed on a 10 mm (length) × 10 mm (width) × 0.5 mm (thickness) silicon substrate and dried at room temperature, then an ultraviolet laser cutting machine LU-5 (HGTECH, Power of 5 W, Wavelength of 355 nm, Huagong Tech. Ltd, Wuhan, China) was used to fabricate microgrooves on the silicon substrate. The laser cutting machine was operated at the speed of 2000 mm/s, with the frequency of 100 kHz, the pulse width of 1 µs, and the current of 1 A. The width of the fabricated grooves was in the range of 100 µm–500 µm, the length of the groove was 4 mm, and the depth of the groove was around 35 µm. Microfibers used for the alignment test are glass fibers with a diameter of 13 µm and a length of 3.7 mm–4 mm. Figure 2 shows the glass microfiber and the fabricated grooved silicon substrate. Figure 2a,b represents the microscopic view of the glass fiber, and the diameter of the glass fiber is around 13 µm, which is about one fifth of a human hair in diameter. Figure 2c shows the fabricated grooved silicon substrate, where the size of the grooves is 4 mm (length) × 0.5 mm (width) × 0.035 mm (thickness). Figure 2d shows an image of the water contact angle (155°) on the superhydrophobic substrate. Figure 2e shows that the water contact angle in the groove is less than 5°.

### 2.3. Experimental Setup

**Figure 3:**
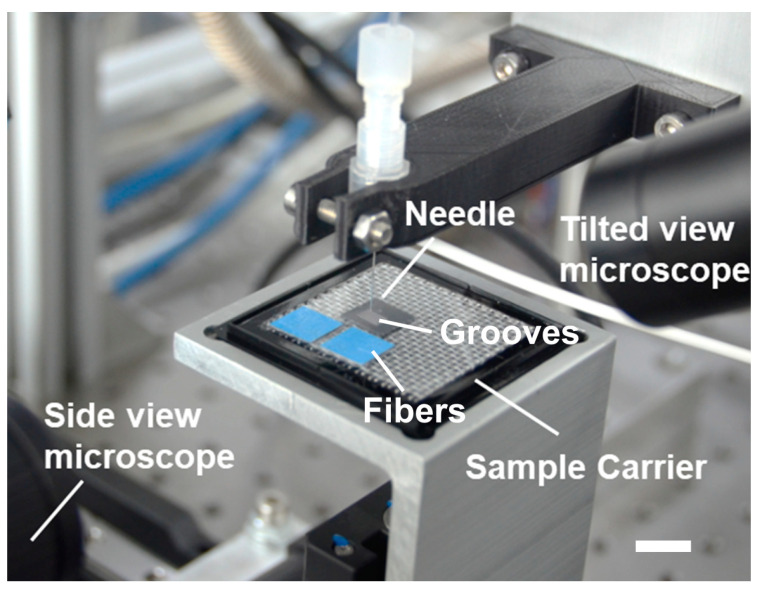
Robotic system for surface tension-based alignment of microfibers on hydrophilic–superhydrophobic grooves. Scale bar: 10 mm.

To align microfibers using surface tension-based alignment method, a robotic system has been set up as shown in Figure 3. The system consisted of a vision system, a needle dispensing system, and a sample carrier. The alignment process was observed both from the tilted view and from the side with a vision system consisting of two microscopes with cameras (Point Grey BFLY-U3-23SS6C and Point Grey GS3-U3-23S6M-C, Edmund Optics, Barrington, IL, USA). The needle dispensing system was attached to a Cavro Centris pump (Tecan Group Ltd, Männedof, Switzerlan) and used to generate nanoliter water droplets on the hydrophilic–superhydrophobic grooved surfaces. The inner diameter of the needle was 160 µm. The sample carrier was built by combining three motorized stages (two M-122.2DD1 and one M-414.3PD by Physik Instrumente, Karlsruhe, Germany), which allowed movement in XYZ-directions. The sample was moved with a controller that had a sequence programmed to its buttons ensuring a constant and repeatable chain of actions.

## 3. Results

### 3.1. Simulations

**Figure 4:**
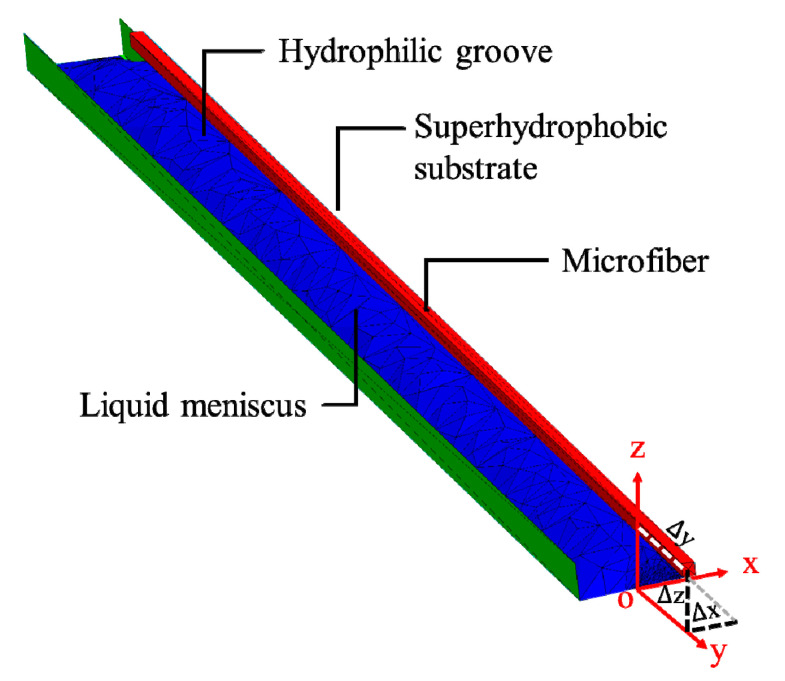
Surface Evolver model of the alignment of microfiber on a hydrophilic–superhydrophobic groove. The model includes three key elements: microfiber, hydrophilic–superhydrophobic groove, liquid meniscus.

Surface tension-based alignment is based on the principle of surface energy minimization of the liquid medium, where the gradient of potential drives the microfiber to align with the microgrooves. To model the alignment process, we used Surface Evolver [32] to find the static equilibrium for the liquid medium by evolving the surface of the liquid using the gradient descent method. Surface Evolver breaks the surface of the liquid droplet into smaller elements, and minimizes the surface energy of each element, by optimizing the location of each vertex. Figure 4 illustrates the simulation of the alignment of microfiber on a hydrophilic–superhydrophobic groove. In the simulation, the size of the fiber is set to be 13 µm in width and 4 mm in length, which matches the size of the glass fiber used in the experiments. The contact angle of the fiber is set to be 15° because the fibers used in our experiments are made of glass, and the measured water contact angle on a glass substrate is 15°. The surface tension of the water is 72.8 mN/m, and the volume of the droplet is set to be 10 nL which is the same as the volume used in the tests. The gravitational force of the glass fiber can be neglected because the size of the microfiber (13 μm) is much smaller than the capillary length (2.7 mm) for water at standard temperature and pressure. The position difference between the release position of the fiber and the center of the groove is defined as the bias, which includes the bias in x-axis labeled as ∆*x*, the bias in y-axis labeled as ∆*y* and the bias in z-axis labeled as ∆*z* as shown in Figure 4. The center of the groove is labeled as “o” in Figure 4.

The restoring force that drives the microfiber to align with the groove can be calculated by:

$$\vec{F}\left(\Delta x,\Delta y,\Delta z\right)=-\nabla E\left(\Delta x,\Delta y,\Delta z\right)$$

where *E* is the surface energy of the liquid meniscus, and ∆*x*, ∆*y*, and ∆*z* are the biases along corresponding axes.

**Figure 5:**
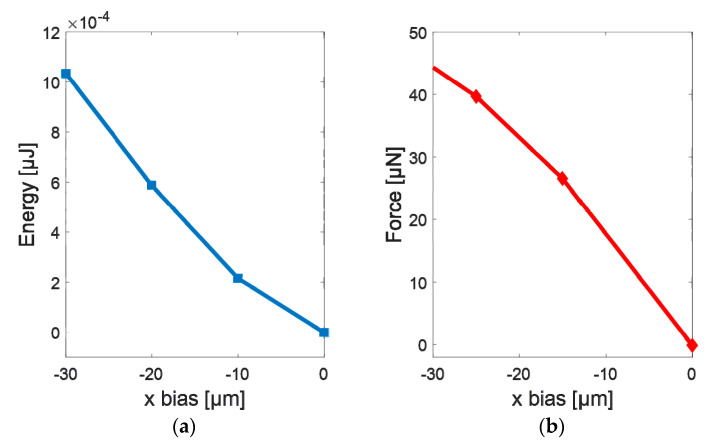
Surface energy and restoring force as the function of x-bias: (**a**) Surface energy of the water meniscus versus x-bias; (**b**) Restoring force driving alignment of microfiber on a hydrophilic–superhydrophobic groove versus x-bias.

Figure 5 shows the surface energy and the restoring force of the water meniscus as the function of the x-bias. The volume of the droplet was kept as 10 nL for all the simulations. The contact angle of water on the substrate was fixed at 155°, which is the same as the measured water contact angle of the superhydrophobic substrate in experiments. The contact angle in the groove was set to be 5° which is the same as the estimation based on the measured contact angle of a water droplet in the groove. Figure 5a shows the relation between the surface energy and the x-bias. The simulation results show that the surface energy of the water meniscus decreases as the fiber moves from its releasing position towards the center of the groove. The surface energy is minimized, and the water meniscus reaches its equilibrium state when the microfiber is aligned with the groove. Figure 5b shows the restoring force as the function of x-bias. The simulation indicates the restoring force decreases as the bias decreases and the restoring force decreases to zero when the bias reaches zero, where the fiber is perfectly aligned with the groove.

**Figure 6:**
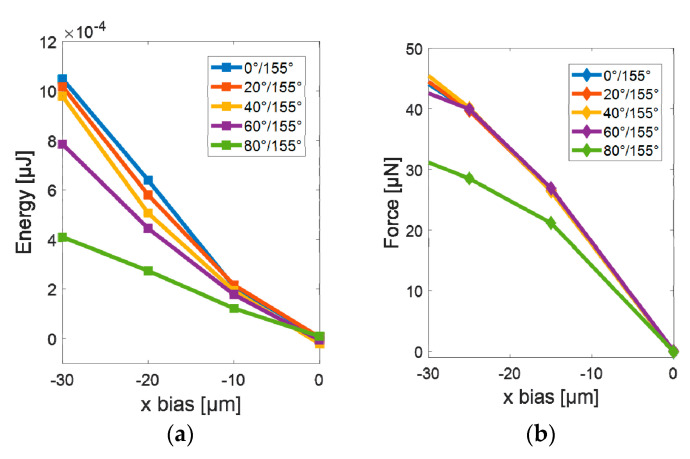
Surface energy and the restoring force as the function of x bias regarding different wetting contrast of grooves: (**a**) Surface energy versus x-bias with a contact angle of the groove from 0° to 80°; (**b**) Restoring force versus x-bias with a contact angle of the groove from 0° to 80°.

To understand the influence of the wetting property of the groove on the alignment process, 5 sets of contact angles were simulated. The water contact angle of the groove varied from 0° to 80°. The contact angle of water on the substrate was kept the same as 155° for all the simulations. Figure 6 shows the surface energy of the water meniscus as the function of x-bias regarding different contact angles in the groove. The simulation indicates that the more hydrophobic the groove is, the more likely the alignment will fail. The reason is that the larger the contact angle is, the smaller the wetting contrast between the groove and the substrate, the flatter the energy curve (Figure 6a) becomes, and the flat energy curve leads to low restoring force for the alignment as shown in Figure 6b.

### 3.2. Experimental Results

**Figure 7:**
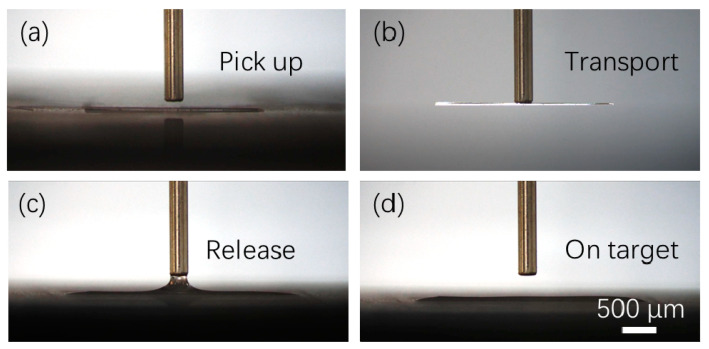
T Sequences of surface tension-based alignment of a glass fiber: (**a**,**b**) The needle picks up a microfiber and transport it to the target groove; (**c**) The needle generates a 10 nL water droplet and the droplet is in contact with the groove forming a water meniscus; (**d**) The water in the groove evaporates and the fiber is aligned with the groove.

Series of experiments were carried out to investigate the proposed surface tension-based alignment method for microfibers. Figure 7 shows the sequences of picking up and releasing a 13 µm wide and 4 mm long glass fiber. Firstly, the fiber is moved below a needle (Figure 7a); and next, the fiber is picked up by the needle (Figure 7b); after that, the fiber is transported to a 100 µm wide and 4 mm long hydrophilic-superhydrophobic groove; then, the needle generates a 10 nL water droplet and the droplet is in contact with the hydrophilic groove with a water contact angle of 5° forming a water meniscus (Figure 7c); finally, the fiber is released on the target groove and aligned with the groove (Figure 7d).

Surface tension-based alignment tests with different volume of the droplet (1 nL to 40 nL) were carried out to find out the relationship between the volume and the success rate. In the experiments, the size of the groove is 4 mm (length) × 0.1 mm (width) × 0.035 mm (thickness). The size of the glass fiber is 4 mm (length) × 0.013 mm (diameter). A successful alignment refers to the fiber being aligned to the groove. Each test was repeated 5 times. The results were summarized in Table 1.

Table 1 shows that the alignment process is very robust to the volume of the droplet and the alignment is reliable with the volume of droplet ranging from 10 nL to 40 nL. When the volume of the droplet is less than 5 nL, the alignment starts to fail mainly due to not enough liquid covering the groove which leads to dry contact and large friction force between the partially wetted groove and the fiber. The upper boundary of the volume is mainly determined by the amount of liquid that can be confined in the groove without spreading. In our case, the superhydrophobic coating plays a very import role in liquid confinement, and the liquid was well confined inside the groove until it reaches to 100 nL.

We also carried out tests to study the influence of the bias on the alignment success rate. The size of the grooves used in the experiment is 4 mm (length) × 0.5 mm (width) × 0.035 mm (thickness). The size of the fiber is 4 mm (length) × 0.013 mm (diameter). Table 2 summarizes the results. The bias in Table 2 refers to the bias in x-axis as defined in Figure 4. The bias is ranging from 250 µm to 50 µm, the volume of the droplet was kept the same (40 nL) for all the tests, and each test was repeated 5 times. The results indicate that the bias has little influence on the alignment process regarding the alignment success rate.

**Figure 8:**
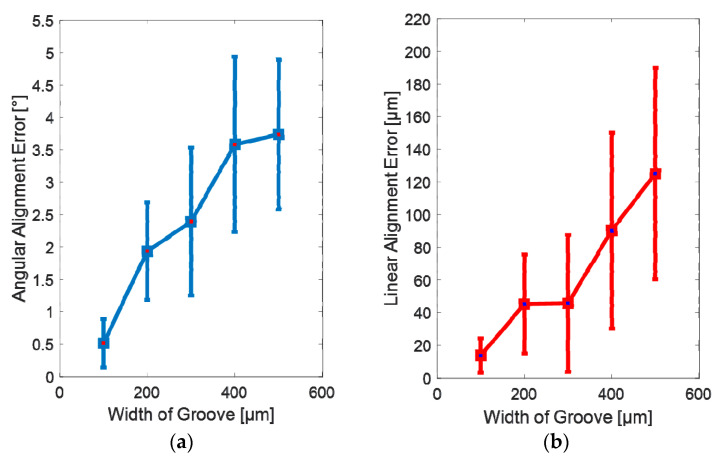
The influence of the width of the groove on the alignment accuracy: (**a**) Angular alignment error as the function of the width of the groove; (**b**) Linear alignment error as the function of the width of the groove.

We have further investigated the relationship between the alignment accuracy and the width of the groove. Alignment accuracy can be described with the linear alignment error and angular alignment error. Linear alignment error is defined as the linear position difference between the final position of the fiber and the center of the groove labeled as “o” shown in Figure 4. Angular alignment error refers to the angular difference between the final position of the fiber and the edge of the groove. The width of the groove used in the experiments varied from 100 µm to 500 µm. 10 nL of water is used in all the tests. Each test has been repeated at least 5 times. Figure 8a shows the angular alignment error as the function of the width of the groove. The x-axis represents the width of the groove, and the y-axis represents the angular alignment error which consists of a mean of 5 repetitions with the standard derivation. The results indicate that the angular alignment error decreases as the width of the groove decreases. When the width of the groove is 100 µm wide, the angular alignment error is less than 1°. It appears that the closer the width of the groove matches the diameter of the fiber, the better the fiber is parallel to the edge of the groove. Figure 8b shows the linear alignment error as the function of the width of the groove. The results indicate that the linear alignment error decreases as the width of the groove decreases. When the groove is 100 µm wide, the linear alignment error is around 15 µm. It appears that the closer the width of the groove matches the diameter of the fiber, the smaller the linear alignment error is.

**Figure 9:**
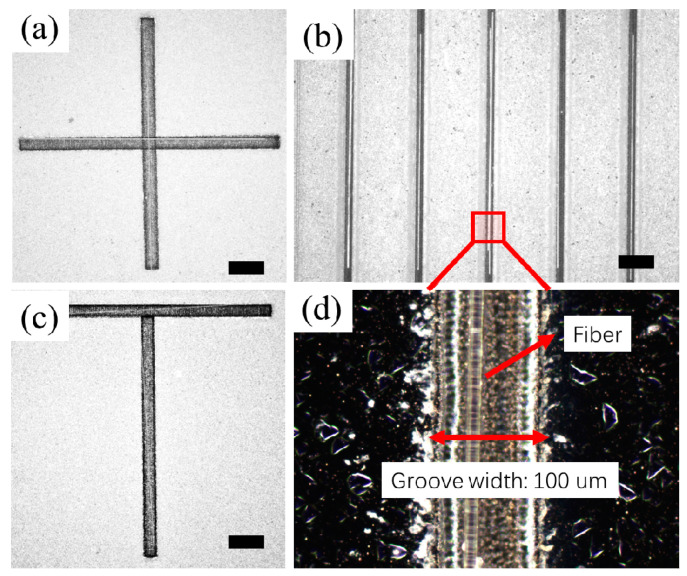
Demonstration of orderly distribution and alignment of microfibers on hydrophilic–superhydrophobic grooves: (**a**) Cross-shape distribution; (**b**) Parallel distribution; (**c**) T-shape distribution; (**d**) Zoomed image of a 100 µm wide groove with a 13 µm fiber inside. Scale bar: 400 µm. (Supplementary Video S1).

To demonstrate the proposed surface tension-based alignment method can be used to construct more complex patterns and geometries with microfibers, we have fabricated hydrophilic–superhydrophobic grooves with different geometries, including T-shape, cross-shape, and parallel line-shape, and carried out the alignment tests. With the proposed method, the glass fibers can be distributed into a certain pattern according to the geometry of the hydrophilic–superhydrophobic grooves as shown in Figure 9.

The results show that microfibers can be distributed orthogonally (Figure 9a–c) and in parallel (Figure 9b). Figure 9d represents a zoomed image of a 13 µm fiber being successfully aligned to a 100 µm wide groove. A demonstration of the surface-tension based alignment of microfibers on the parallel hydrophilic–superhydrophobic grooved surface is shown in the supplementary video (Supplementary Video S1).

**Figure 10:**
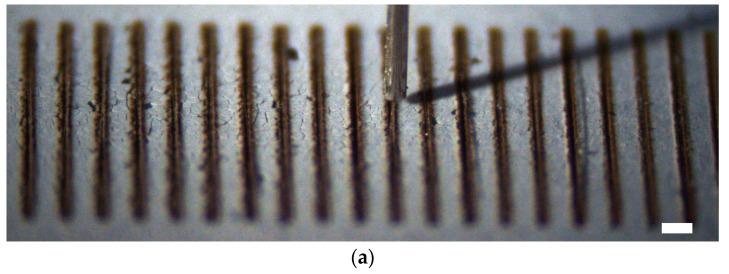

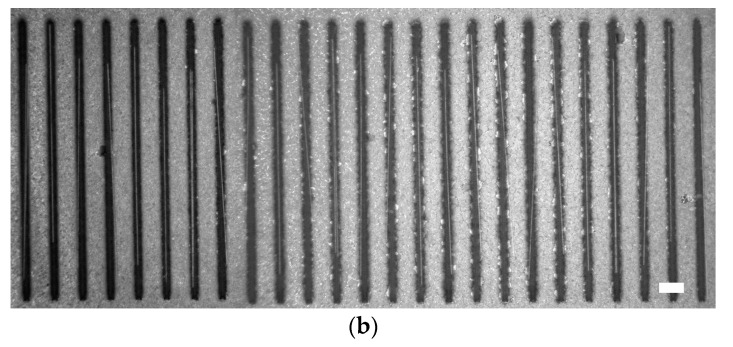
Alignment of 25 microfibers to parallel grooves: (**a**) Titled view of a fiber released to the corresponding hydrophilic–superhydrophobic groove; (**b**) Top view of 25 fibers aligned to the corresponding grooves. Scale bar: 200 µm.

Figure 10 shows that 25 microfibers with a diameter of 13 µm have been successfully aligned to its corresponding grooves with a width of 100 µm. This shows that the proposed surface tension-based alignment method has great potential to be applied to construct complex fiber-based microstructures.

## 4. Discussions and Conclusions

To sum up, the alignment accuracy is largely depending on the size difference between the groove and the microfiber, the better the size of the groove matches the size of the fiber, the higher the alignment accuracy will be achieved. In our experiments, the grooves were fabricated using an ultraviolet laser cutting machine and the minimum spot size is approximately 50 µm, which limited the capability of fabricating grooves with the width less than 100 µm. The width of the groove could be further decreased to several to tens of micrometers using a picosecond laser cutting technique to match the size of the microfiber. The proposed alignment methods will be extended by using sacrificial substrate for potential applications in fabrication of advanced fiber-enhanced functional materials. In our experiments, we used glass fibers, but this proposed surface tension-based alignment method might be also applied to the microfibers of other materials. Although we only tested water as the medium for the alignment, other low surface tension liquid may also work if the liquid can be confined inside the groove. The alignment with water normally takes tens of milliseconds, regarding to the liquid medium of higher evaporation rate, such as ethanol, if the evaporation of the liquid is slower than the alignment, the alignment should also work.

This paper proposes a surface tension-based method to align and orderly distribute microfibers. A theoretical model was developed to simulate the alignment of the microfiber and analyze the driven force for the alignment of microfibers. The simulation shows the larger the wetting contrast of the groove, the larger the restoring force for alignment. We designed and fabricated hydrophilic–superhydrophobic grooved surfaces. We demonstrated that the microfibers can self-align to the hydrophilic–superhydrophobic grooves with different geometries. We further studied the influence of the volume of the droplet and the bias on the alignment success rate. The results indicate that the process can tolerate large variations of the bias and the volume unless the volume is not enough to cover the groove. Furthermore, we investigated the influence of the width of the grooves on the alignment accuracy and verified that the alignment accuracy increases as the width of the groove decrease. The proposed method has great potential in construction of complex microstructures using microfibers.

## Figures and Tables

**Table 1:** Influence of volume of droplet on alignment.

Volume [nL]	Success Rate
40	100%
30	100%
20	100%
10	100%
5	80%
1	0%

**Table 2:** Influence of bias on alignment.

Bias [µm]	Success Rate
250	100%
200	100%
150	100%
100	100%
50	100%

## Supplementary Materials

The following are available online at https://www.mdpi.com/2072-666X/11/11/973/s1, Video S1: Surface Tension-based Alignment of Microfibers on Hydrophilic-superhydrophobic Grooved Surface.
